# Adolescent Life Satisfaction Explained by Social Support, Emotion Regulation, and Resilience

**DOI:** 10.3389/fpsyg.2021.694183

**Published:** 2021-09-23

**Authors:** Lorea Azpiazu Izaguirre, Arantzazu Rodríguez Fernández, Eider Goñi Palacios

**Affiliations:** ^1^Department of Developmental and Educational Psychology, Faculty of Education, Philosophy and Anthropology, University of the Basque Country (UPV/EHU), Donostia-San Sebastian, Spain; ^2^Department of Developmental and Educational Psychology, Faculty of Education and Sport, University of the Basque Country (UPV/EHU), Vitoria-Gasteiz, Spain

**Keywords:** social support, emotional regulation, resilience, satisfaction with life, adolescence, cross-sectional survey

## Abstract

Adolescence is a stage characterized by many biological and psychosocial changes, all of which may result in a decrease in subjective well-being. It is therefore necessary to identify those factors that contribute to increased life satisfaction, in order to promote positive development among young people. The aim of this study is to examine the dynamics of a set of variables that contribute to life satisfaction. A total of 1,188 adolescents (aged between 12 and 16 years) completed the Perceived Social Support from Family and Friends and Perception of the School Environment Questionnaires, the Trait Meta Mood Scale (TMMS), Connor-Davidson Resilience Scale-10 (CD-RISC), and Satisfaction with Life Scale (SWLS) for social support, emotion regulation, resilience, and life satisfaction. By applying structural equation modeling (SEM), the results reveal a direct prediction of family support, emotion regulation, and resilience on life satisfaction. Support from friends and emotion regulation was also found to explain resilience, and support from family and teachers was found to predict emotion regulation. In conclusion, emotion regulation and social support were found to indirectly affect life satisfaction among adolescents through resilience. The theoretical and practical implications of these results are discussed.

## Introduction

Positive psychology aims to identify the strengths that foster healthy development and the optimal environments that foster physical and psychological well-being (Compton and Hoffman, 2019). Within this approach, considerable attention has been paid to subjective well-being, identified as a key driver of development (Steinmayr et al., 2019). Given that some studies have found a notable decrease in well-being during adolescence (González-Carrasco et al., 2017; Orben et al., 2020), as a result of the biological and psychosocial changes associated with this life stage (Morrish et al., 2019), it is vital to identify the factors that boost *life satisfaction*, which, according to Diener et al. (2017), is a key cognitive dimension of hedonic subjective well-being.

Although a fair amount of research has been carried out in this field with adults, less attention has been paid to adolescents (Tian et al., 2015). Therefore, identifying the factors that contribute to life satisfaction will help promote positive development during adolescence. In this regard, the research shows that psychological assets such as emotion regulation and resilience influence how individuals in this age group assess their life satisfaction (Cejudo et al., 2016).

Resilience is considered to be particularly complex, as has been studied from difference perspectives so includes traits, outcomes, and processes related to the ability to overcome from unfavorable circumstances (Oshio et al., 2018). Despite this discrepancy, it is commonly defined as the ability to overcome adversity, and to recover and gain strength from highly stressful events (Campbell and Stein, 2007; Southwick et al., 2014; Masten, 2018). Although, there is a continuous debate on their feasibility, reliability, and validity in different settings (Kaunda-Khangamwa et al., 2020), the most common way to assess resilience is through self-report measures such as the Connor-Davidson Resilience Scale (Song et al., 2020). Resilience is widely believed to have a positive effect on life satisfaction (Arslan, 2019; Ramos-Díaz et al., 2019; Yang et al., 2020), which is why it is considered an important source of subjective well-being (Yildirim and Belen, 2019). It has also been associated with life satisfaction in conjunction with *emotion regulation*, understood as the ability to cope with one’s own and others’ feelings in terms of how they are perceived, assessed, experienced, or expressed (Gross, 2014; McRae and Gross, 2020). Emotional regulation includes processes and outcomes related to emotional management (Sarrionandia et al., 2015). It is considered a central topic in adolescents’ positive developmental perspective (Morrish et al., 2018) and a relevant ability included in the emotional intelligence model of Mayer and Salovey (1997). Although very few studies focus on all three variables at once, the few that do report significant correlations between emotion regulation, resilience, and life satisfaction, with the impact of emotion regulation on life satisfaction being mediated by resilience (Cejudo et al., 2016; Ramos-Díaz et al., 2019; Wu et al., 2021).

Another variable associated with life satisfaction is perceived social support (Diener et al., 2018), understood as an individual’s subjective perception that they are cared for, loved, and a member of a network of mutual obligations (Cobb, 1976). However, it is unclear whether this relationship is a direct (Rodríguez-Fernández et al., 2012; You et al., 2018) or indirect one, mediated by individual variables (Vieno et al., 2007; Rodríguez-Fernández et al., 2016a) such as resilience or emotion regulation (You et al., 2018; Yıldırım and Çelik, 2020). It is widely accepted that social support is one of the most crucial factors for coping with and overcoming difficulties (Southwick et al., 2016; Arslan, 2019; Yang et al., 2020; Yıldırım and Çelik, 2020), as well as for developing good emotion regulation (Azpiazu et al., 2015; You et al., 2018; Yıldırım and Çelik, 2020; Gómez-Ortiz et al., 2019). Research has also shown that there are correlations between the three sources of support (family, friends, and teachers) and emotion regulation, resilience, and life satisfaction (Siddall et al., 2013; Azpiazu et al., 2015; Rodríguez-Fernández et al., 2016a; Ramos-Díaz et al., 2019; Geng et al., 2020). However, few studies have attempted to untangle the intricate web of relationships that exist between these three variables (social support, emotion regulation, and resilience) in terms of their impact on life satisfaction.

The ecological system (Bronfenbrenner and Morris, 2007) and other previous studies argue that psychological assets such as emotion regulation (You et al., 2018) and resilience (Rodríguez-Fernández et al., 2016a; Geng et al., 2020; Yang et al., 2020) mediate the relationship between the different types of social support and life satisfaction, and that the influence of social support may differ depending on the specific source of support analyzed (You et al., 2018). Some identify family support (more than support from friends or teachers) as being fundamental to both adolescents’ positive development (Rueger et al., 2010; Siddall et al., 2013) and their emotion regulation, although in this last case, similar levels of significant prediction have also been found for support from friends and teachers (Azpiazu et al., 2015). Other authors argue that the family is the only source of support that predicts emotion regulation, resilience, and life satisfaction, with that provided by friends and teachers not being significant at all (Rodríguez-Fernández et al., 2016b; Fernández-Lasarte et al., 2019). Finally, a third group of authors identify the support from friends as being an important predictor of resilience (Park and Park, 2020), even more so than family support (Van Harmelen et al., 2017; Chen, 2019), arguing that this type of support also influences (albeit weakly) life satisfaction (Rodríguez-Fernández et al., 2012). In sum, although there is general agreement regarding the fact that individual psychological variables mediate the relationship between social support and life satisfaction, disagreement exists regarding both which sources of support are the most influential and the intensity of their impact.

When attempting to explore this intricate, complex web of multivariate relationships, we must also take into account those studies that claim there is a link between the three types of social support. In other words, perceptions regarding one source of support are viewed as influencing the rest, since, for example, interactions in the family environment may foster or hinder the development of the abilities required for building positive relationships in other domains, and may therefore influence the perception of support provided by friends or teachers (Moore et al., 2018).

**Figure 1:**
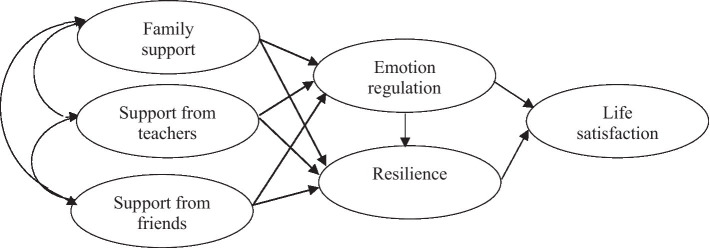
Proposed theoretical model.

The present study is relevant, firstly, because empirically test a theoretical model of life satisfaction by analyzing the associations between different types of perceived social support and life satisfaction, explained by emotion regulation and resilience (Figure 1); and secondly, because it aims to determine which contextual factor has the greatest prediction on psychological assets and life satisfaction, since differentiating between types of social support in accordance with source tends to provide more specific information about adolescent development (Compton and Hoffman, 2019). Achieving these aims will enable us to (a) expand existing scientific knowledge, since it will help redress the lack of studies analyzing the relationships which exist between these variables, and contribute to clarifying the heterogeneity of the results reported to date (You et al., 2018), and (b) identify those factors that contribute to life satisfaction, with the aim of fostering adolescent well-being in a life stage in which a notable drop in subjective well-being has been observed. If the aim is to ensure psychologically well-adjusted young people, then this decrease in well-being must be addressed (González-Carrasco et al., 2017; Orben et al., 2020).

## Materials and Methods

### Participants

Participants were 1,188 secondary school students (546 girls and 642 boys) aged between 12 and 16years (*M*=14.24; *SD*=1.0), selected using the incidental sampling method. Of the total sample, 690 (58.1%) came from public schools and 498 (49.1%) from semi-private schools (i.e., private schools that also receive some state funding) in the Autonomous Community of the Basque Country (Spain). In terms of academic level, 670 (54.6%) were in the first 2years of compulsory secondary education and 518 (43.6%) in the second 2years.

### Instruments

*Family support* and *support from friends* were measured using the *Cuestionario de Apoyo Social percibido de Familia y Amigos* (Perceived Social Support from Family and Friends Questionnaire; AFA; González-Ramírez and Landero, 2014), the two dimensions of which correspond to the two types of support measured. The instrument has a five-point Likert-type response scale ranging from (1) *never* to (5) *always*. *Family support* comprises eight items (“someone in your family supports you when you are at school”) and *support from friends* comprises seven items (“you are satisfied with the support you receive from your friends”). Item 10 was removed due its low factor loading (*β*=0.289, *R*^2^=0.084) and the fact that the authors of the scale themselves identify it as controversial. The fit indexes for the *family support* measure were: *χ*^2^_[df]_=58_[12]_, TLI=0.964, CFI=0.971, IFI=0.980, RMSEA =0.057 (0.043–0.072), SRMR=0.028, *a*=0.854, AVE=0.487, and CRC=0.868; and for *support from friends*: *χ*^2^_[df]_=36_[9]_, TLI=0.967, CFI=0.980, IFI=0.981, RMSEA =0.050 (0.034–0.068), SRMR=0.023, *a*=0.856, AVE=0.517, and CRC=0.864.

To measure *support from teachers*, we used the eight-item teacher support subscale of the Spanish adaptation by Moreno et al. (2012) of the *Percepción del Entorno Escolar* (Perception of the School Environment) questionnaire (HBSC; 2006). The scale comprises eight items (“our teachers are kind and friendly”) and has a five-point Likert-type response scale ranging from (1) *totally agree* to (5) *totally disagree*. The goodness of fit indexes for this study was: *χ*^2^_[df]_=139_[20]_, TLI=0.931, CFI=0.951, IFI=0.951, RMSEA =0.071 (0.060–0.082), SRMR=0.036, *a*=0.840, AVE=0.416, and CRC=0.849.

*Emotion regulation* was measured using the eight-item subscale of the *Trait Meta Mood Scale-24* (TMMS-24; Salovey et al., 1995; Fernández-Berrocal et al., 2004), rated on a Likert-type scale from 1 (*strongly disagree*) to 5 (*strongly agree*). It includes items such as, “although I am sometimes sad, I have a mostly optimistic outlook.” Item 23 was eliminated due to its low factor loading (*β*=0.337, *R*^2^=0.114; Antonio-Agirre et al., 2019). The fit indexes obtained in this study were: *χ*^2^_[df]_=90_[14]_, TLI=0.960, CFI=0.973, IFI=0.973, RMSEA =0.067 (0.054–0.081), SRMR=0.043, *a*=0.843, AVE=0.452, and CRC=0.845.

*Resilience* was measured using the *Connor-Davidson Resilience Scale-10* (CD-RISC; Campbell and Stein, 2007; Notario et al., 2014). This instrument comprises 10 self-report items, each rated on a Likert-type scale from 0 (*not true at all*) to 4 (*true nearly all the time*). In its original version, the 10 items load on a single dimension (“I am able to adapt to change”). Items 8 (*β*=0.351, *R*^2^=0.123), 19 (*β*=0.345, *R*^2^=0.119), and 7 (*β*=0.359, *R*^2^=0.129) were eliminated due to their low factor loadings (Serrano-Parra et al., 2012; Gras et al., 2019), and the fit indexes for this study were: χ^2^_[df]_=54_[14]_, TLI=0.942, CFI=0.961, IFI=0.961, RMSEA =0.049 (0.039–0.063), SRMR=0.032, *a*=0.727, AVE=0.285, and CRC=0.733.

*Life satisfaction* was measured using the *Satisfaction with Life Scale* (SWLS; Diener et al., 1985; Atienza et al., 2000). Each item is answered on a seven-point Likert-type scale ranging from 1 (*strongly disagree*) to 7 (*strongly agree*). It includes items such as, “in most ways, my life is close to my ideal.” The fit indexes for this study were: *χ*^2^_[df]_=22_[5]_, TLI=0.972 CFI=0.986, IFI=0.986, RMSEA =0.054 (0.032–0.078), SRMR=0.022, *a*=0.822, AVE=0.510, and CRC=0.837.

### Procedure

We contacted the principals and deputy principals of the participating schools to inform them of the study aims and request their authorization and the informed consent of parents. Data were collected from each class in sessions lasting approximately 40min. To reduce social desirability bias and insincere answers, students were told that their participation was strictly voluntary. The instruments were administered collectively in the children’s classrooms by members of the research team. The single blind criterion was followed, thus preventing participants from knowing the aim of the research study being carried out. The study complies with the ethical criteria established by the University of the Basque Country.

### Data Analysis

The univariate and multivariate normality of the data were analyzed. The KS test and Mardia’s coefficient (*Mardia*=590.65, *Z*=175.60) indicated the absence of normality in both cases. We therefore opted for robust indexes, using the EQS 6.3 program. Nevertheless, parametric procedures were also employed using the SPSS 22 program, since the results remain robust in the event of the normality assumption not being met (Montilla and Kromrey, 2010).

To estimate the measurement and structural model, we used the structural equation modeling (SEM) method in accordance with the criteria established by Byrne (2006), analyzing the residual covariance matrix and interpreting a combination of the most commonly used fit indexes (McDonald and Ho, 2002): *χ*2/df≤5.0; CFI, TLI, and IFI≥0.90; RMSEA≤0.08 and their CIs (90%); and SRMR≤0.08. We used the Chi-squared test to test differences in the model fit. We also calculated the AIC and CAIC comparative indexes, considering the model with the lowest values to be the most parsimonious (West et al., 2014).

## Results

### Global Fit of the Proposed Model

**Figure 2:**
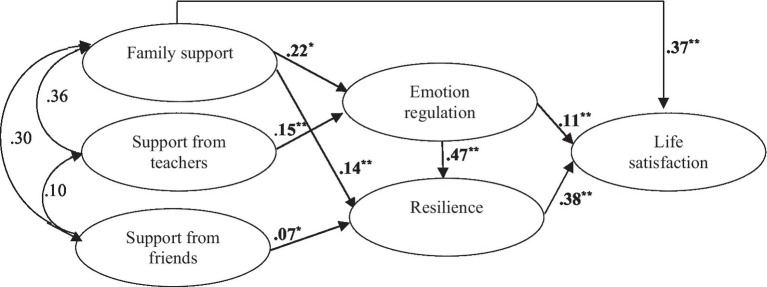
Standardized solution for the structural model; ^*^p<0.05, ^**^p<0.01.

After checking the significance of the inter-variable correlations (Table 1) and the suitability of the measurement model (*χ*^2^_[df]_=1941_[723]_, CFI=0.915, TLI=0.908, IFI=0.922, RMSEA =0.038 (0.036–0.040), and SRMR=0.043), we tested the hypothesized model, obtaining satisfactory goodness of fit values (Table 2). Two paths failed to reach explanatory significance: *support from friends*-*emotion regulation* (*β*=0.059; *z*=1.550; *p*>0.05) and *support from teachers-resilience* (*β*=0.032; *z*=0.885; *p*>0.05). Moreover, the modification indexes returned by the Wtest suggested it would be best to eliminate those relationships. The LMtest suggested including a path between *family support* and *life satisfaction*, an association that is theoretically justified (You et al., 2018). The new indexes were better than in the initial model (Table 2). The results of the Chi-squared test of discrepancy were significant (*Δχ^2^*=92.12, *p*<0.001), indicating the greater suitability of the new model in comparison with the initial one. The final model also had a smaller AIC and CAIC, indicating a better fit. In conclusion, the results revealed a good fit of the final model to the data (Figure 2), with *family support*, *emotion regulation*, and *resilience* explaining 43.4% of the variance observed in *life satisfaction*.

**Table 1:** Means and correlations.

S.no	Variables	1	2	3	4	5	*M*(*SD*)
1.	Family support	-	-		-		32.56 (5.33)
2.	Support from teachers	0.248^**^	-	-	-		25.75 (5.82)
3.	Support from friends	0.352^**^	0.116^**^	-	-		28.48 (4.41)
4.	Emotion regulation	0.232^**^	0.201^**^	0.143^**^	-		27.23 (6.70)
5.	Resilience	0.257^**^	0.150^**^	0.166^**^	0.488^**^		30.30 (4.24)
6.	Life satisfaction	0.392^**^	0.229^**^	0.219^**^	0.366^**^	0.458^**^	25.91 (5.63)

* *p* <0.05;

** *p* <0.01

**Table 2:** Goodness of fit.

Models		*χ*^2^/df	CFI	TLI	IFI	RMSEA (90% IC)	SRMR	AIC	CAIC
Initial	2064.76_[726]_	2.84	0.906	0.899	0.907	0.039 (0.037–0.041)	0.056	612.76	−3801.341
Final	1956.84_[727]_	2.69	0.914	0.908	0.914	0.038 (0.036–0.040)	0.045	502.84	−3917.34

### Direct and Indirect Effects Between the Study Variables

Figure 2 shows the estimated structural model with standardized coefficients and their associated probability. An analysis of these coefficients indicated a correlation between the three types of support and their differential effect on psychological assets. Only *family support* was found to have a direct prediction on *life satisfaction* (*β_d_*=0.37; *z*=9.035; *p*<0.01), with this prediction being the highest one observed in relation to this type of support. Although *support from friends* (*β_i_*=0.03; *z*=1.984; *p*<0.05) and *support from teachers* (*β_i_*=0.04; *z*=3.780; *p*<0.01) were found to indirectly predict *life satisfaction*, this prediction was relatively weak (Table 3).

**Table 3:** Direct and indirect effects.

	*β*	*z*
Direct effects		
Family support→emotion regulation	0.220^**^	5.249
Support from teachers→emotion regulation	0.148^**^	4.118
Family support→ resilience	0.142^**^	3.173
Support from friends→resilience	0.076^*^	2.018
Emotion regulation→resilience	0.469^**^	10.743
Family support→ life satisfaction	0.367^**^	9.035
Emotion regulation→life satisfaction	0.109^**^	2.794
Resilience→life satisfaction	0.378^**^	8.488
Indirect effects		
Family support→emotion regulation→resilience	0.103^**^	4.806
Support from teachers→emotion regulation→resilience	0.069^**^	3.803
Family support→emotion regulation→resilience→life satisfaction	0.116^**^	4.459
Support from friends→emotion regulation→resilience→life satisfaction	0.029^*^	1.984
Support from teachers→emotion regulation→resilience→life satisfaction	0.042^**^	3.780
Emotion regulation→ resilience →life satisfaction	0.177^**^	6.950

* *p* <0.05, *z*=1.96;

** *p* <0.05, *z*=2.56;

The highest standardized coefficient found in all the relationships studied corresponded to the *emotion regulation* and *resilience* association (*β_d_*=0.47; *z*=10.743; *p*<0.01). *Resilience* was found to be associated with all the variables studied, with its relation with *emotional regulation* and *life satisfaction* being particularly striking, since the indirect prediction (*β_i_*=0.18; *z*=6.950; *p*<0.01) of *emotional regulation* on *satisfaction with life* was greater than the direct one (*β_d_*=0.11; *z*=2.794; *p*<0.01). It is also worth highlighting that the direct prediction of *emotion regulation* was weaker than that of both *family support* (*β_d_*=0.37; *z*=9.035; *p*<0.01) and *resilience* (*β_d_*=0.38; *z*=8.488; *p*<0.01). The three types of support explained 9% of the variance observed in *emotion regulation*, and, together with *emotion regulation*, explained 30% of the variance observed in *resilience*.

## Discussion and Conclusion

Much of the psychological research interested in the study of well-being in the adolescent stage has been devoted mainly to the identification and way of remedying adolescent deficits, oriented, above all, to the study of clinical problems (Proctor et al., 2017). However, with the emergence of positive psychology, the focus of attention has changed (Lomas et al., 2020), deriving the interest of studies toward the identification of the strengths that promote healthy development, as well as to the creation of optimal environments that favor physical and psychological well-being (Compton and Hoffman, 2019). Within this current of study, life satisfaction receives considerable attention as is one of the most well-established indicators of well-being and positive functioning among young people (Diener et al., 2017).

Thus, the aim of this study was to empirically test a theoretical model of life satisfaction in order to analyze the psychological assets that foster positive development during adolescence, a life stage characterized by a drop in psychosocial adjustment and subjective well-being (Morrish et al., 2019). The specific aim was to determine the nature of the system of relationships, which exists between individual and contextual variables in terms of their prediction on life satisfaction.

One of the variables analyzed was social support, particularly in terms of its prediction on emotion regulation and resilience, with statistically significant results being found for both psychological assets in accordance with the specific type of support in question. This is consistent with that reported by previous studies, which found that, although social support is a key correlate for adolescent psychological development, according to Southwick et al. (2016), it is not universally useful, since its effectiveness varies in accordance with the source and type of the support provided and the degree to which it meets the individual’s specific needs. In the present study, family support was found to be the most important (in terms of both degree and areas of influence), having a particularly strong prediction on emotion regulation, although also (to a lesser extent) on resilience. For its part, support from teachers was only found to influence emotion regulation, and a weak yet significant prediction of support from friends on resilience was also observed. Some previous studies identify the family as the only important source of support (Siddall et al., 2013; Rodríguez-Fernández et al., 2016b; Fernández-Lasarte et al., 2019), while others, consistently with that found here, agree that family is important, but also argue that support from friends and teachers is a relevant factor to take into consideration (Azpiazu et al., 2015; Van Harmelen et al., 2017; You et al., 2018).

Although, as stated earlier, the results of the present study indicate that family support is more important than that provided by friends or teachers, they also suggest that only adults explain adolescents’ ability to handle their own emotions, since peer support is the only source that seems to have no prediction on emotion regulation. One possible explanation for this may be found in the meta-analysis carried out by Páez (2004), who argues that support seeking is an instrumental strategy for effectively learning about and regulating emotions. Consequently, having recourse to and feeling supported by adults with more extensive life experience may influence adolescents’ perceptions of their own capacity to regulate and manage their emotions.

As regard resilience, consistently with that reported by previous studies (Masten and Palmer, 2019), our results point to family support as a key factor in helping adolescents overcome difficult situations. There are, however, discrepancies regarding the role played by friends, since the prediction of peer support found here was fairly weak (Rodríguez-Fernández et al., 2012) in comparison with that observed in other studies (Van Harmelen et al., 2017; Chen, 2019). These findings are consistent with the theory in that, in stressful situations, people generally tend to seek advice, favors, protection, and emotional support in their immediate environment (family and friends), whereas support from teachers is associated more with informational aid (Malecki and Demaray, 2003), which does not activate resilience.

For its part, emotion regulation was found to have a direct prediction on life satisfaction (Cejudo et al., 2016; You et al., 2018), as well as an indirect one through resilience (Ramos-Díaz et al., 2019). This indicates that although adolescents’ perception of being able to regulate their emotions may lead how satisfied they feel with their life, the prediction of good emotion regulation is much greater when combined with the capacity for resilience in response to difficult situations. In other words, when faced with a difficult situation, the ability to exercise good emotion regulation, coupled with the capacity for resilience, results in a higher level of life satisfaction. What remains to be clarified, however, is whether this is due to individuals perceiving themselves as capable of overcoming adverse circumstances, which in turn gives rise to greater life satisfaction, or if it is because mobilizing this resilience helps preserve a high initial level of life satisfaction, which would otherwise be diminished.

One novel finding was the partial explanation predicted by both emotion regulation and resilience in relation to life satisfaction. Previous studies have reported varied and even contradictory results, since although some report only an indirect influence, mediated by personal variables (Vieno et al., 2007; Rodríguez-Fernández et al., 2016a), a recent study observed a significant direct association between support and life satisfaction (You et al., 2018). However, findings of this study support, for the first time, the important role of emotion regulation and resilience in explaining the association between social support and life satisfaction, since the data reveal that emotion regulation and resilience also predict positively the direct association between these variables. This has important practical and psychoeducational implications, since it broadens the scope for intervening to improve adolescents’ life satisfaction. For example, psychoeducational programs could be designed to foster the support provided to adolescents by different stakeholders, although they could also be oriented toward combining the best elements of these real sources of support with the capacity for emotion regulation and resilience. Moreover, Casas and Frønes (2020) highlight the importance of including relative notions of subjective well-being in social policies and intervention programs, with the aim of creating synergies to foster positive development.

In relation to previous research, the present study suggests that the social support perceived in each microsystem (family, friends, and teachers) impacts the others to some extent (Bronfenbrenner and Morris, 2007), with the family environment facilitating or hampering the development of the capacities required to build positive relationships, and friends and teachers sometimes compensating for situations of scarce parental support (Moore et al., 2018). However, our results continue to point to the key role played by family support during adolescence, with the prediction of the family environment being greater than that of friends and teachers (Rueger et al., 2010; Siddall et al., 2013). There are two possible explanations for this, both of which are open to debate: (a) despite the autonomy and independence that are so characteristic of this life stage, perceived family support continues to be a significant factor for adolescents, although there is no linear cause-effect relationship that supports the premise “more family contact leads to greater perceived support”; and (b) the support provided by friends is such an important and endogenous aspect of adolescent psychology that it acts more as a moderator of individual variables (Chen, 2019), which explain why, in this study, family support was found to have a greater influence than support from friends.

In summary, this study has demonstrated that contextual and personal factors are important considerations when studying how adolescents feel about their lives (You et al., 2018). Concretely, this study evidenced that feeling loved and supported by family makes adolescents feel good and satisfied and provides various psychological benefits such as greater emotional regulation and resilience that also facilitate the perception of leading satisfying lives. Thus, the results highlight the need to create positive familiar environments. The study also confirmed previous findings (Arslan, 2019; Ramos-Díaz et al., 2019) in the sense that resilience has a decisive prediction on satisfaction, and in less extent, emotional regulation explained by resilience. According to the results, it can be argued that educational environments should be made more active in fostering resilience and emotional intelligence support activities (gratitude, mindfulness, and strengths based activities) in order to promote life satisfaction (Howell et al., 2013).

The present study has several limitations that should be taken into consideration. Firstly, only self-report measures were used. Future research should consider also using objective evaluations, such as the real support provided by the different sources, to complement the analysis of the proposed model. Secondly, as Rueger et al. (2010) point out, it is useful to study the differential impact of the type of support provided: *emotional*, *instrumental*, and *informational* (in terms of its mediating and moderating role), as well as that of the source of said support. Doing this would have provided a more accurate view of the multicausal influence of social support on both psychological assets and life satisfaction. Finally, the cross-sectional nature of the study makes it impossible to demonstrate true dependence between variables. We should therefore make it clear that when we talk here about influence or effect, we are always referring to statistical impact or effect, which must be empirically confirmed in the future using experimental designs.

## Data Availability Statement

The datasets presented in this article are not readily available because the ethics committee that has approved the project does not allow sharing the data with third parties due to the confidentiality signed by the participants. Requests to access the datasets should be directed to lorea.azpiazu@ehu.eus.

## Ethics Statement

This study has received a favorable report from the University of the Basque Country’s Ethics Commission (CEISH/UPV-EHU, BOPV 32, and EHAA, report number M10/2015/076). Written informed consent to participate in this study was provided by the participants’ legal guardian/next of kin.

## Author Contributions

LA, AR-F, and EG conceived the design, drafted the manuscript, and co-led the preparation of the manuscript. LI conducted the data analysis. All authors contributed to the article and approved the submitted version.

## Funding

This research project was funded by the IT1217-19 Consolidated Research Group within the Basque University System, and by the EDU2017-83949-P Project run by the Spanish Ministry of the Economy, Industry and Competitiveness’ State Knowledge Generation Subprogram.

## Conflict of Interest

The authors declare that the research was conducted in the absence of any commercial or financial relationships that could be construed as a potential conflict of interest.

## Publisher’s Note

All claims expressed in this article are solely those of the authors and do not necessarily represent those of their affiliated organizations, or those of the publisher, the editors and the reviewers. Any product that may be evaluated in this article, or claim that may be made by its manufacturer, is not guaranteed or endorsed by the publisher.
